# H-Wave^®^ Device Stimulation for Chronic Knee Pain Disorders: A Patient-Reported Outcome Measures Observational Study

**DOI:** 10.3390/medicina62010075

**Published:** 2025-12-30

**Authors:** Ashim Gupta, David Han, Stephen M. Norwood

**Affiliations:** 1 Future Biologics, Lawrenceville, GA 30043, USA; 2 Department of Management Science and Statistics, The University of Texas at San Antonio, San Antonio, TX 78249, USA; 3 Orthopaedic Surgeon (MD, FAAOS), Austin, TX 78738, USA

**Keywords:** electrical stimulation, chronic pain, knee, knee osteoarthritis, osteoarthritis, H-Wave^®^, H-Wave device stimulation, PROMs, patient-reported outcome measures

## Abstract

*Background and Objectives*: Chronic knee pain (cKP) affects approximately 25% of adults worldwide, with prevalence increasing over recent decades. While conventional treatments have clinical limitations, several types of electrical stimulation have been suggested to improve patients’ quality of life. The electrical stimulation literature contains inadequate patient-reported outcome measures (PROMs) data. Encouraging preliminary H-Wave^®^ device PROMs results for chronic neck, shoulder, and low back pain have previously been published. This PROMs study’s goal is to similarly assess the efficacy of H-Wave^®^ device stimulation (HWDS) in patients with differing knee disorders. *Materials and Methods*: This is an independent, retrospective, observational cohort study analyzing H-Wave^®^ PROMs data, prospectively and sequentially collected over 4 years. In total, 34,192 pain management patient final surveys were screened for participants who were at least 18 years old, used H-Wave^®^ for any knee-related disorder, reporting chronic pain from 90 to 730 days, with device treatment duration from 22 to 365 days. PROMs included effects on function, pain, sleep quality, need for medications, ability to work, and patient satisfaction; additional data includes gender, age (when injured), chronicity of pain, prior treatments, and frequency and length of device use. *Results*: PROMs surveys from 34,192 HWDS patients included 1143 with “all knee”, 985 “knee injury”, and 124 “knee degeneration” diagnoses. Reported improvements in function/ADL (96.51%) and work performance (84.63%) were significant (*p* < 0.0001), with ≥20% pain relief in 86.76% (*p* < 0.0001), improving 2.96 points (average 0–10 NRS). Medication use decreased (69.85%, *p* = 0.0008), while sleep improved (55.33%) in knee injury patients. Patient satisfaction measures exceeded 96% (*p* < 0.0001). Subgroup analysis suggests that longer device use and shorter pain chronicity resulted in increased (*p* < 0.0001) HWDS benefits. *Conclusions*: HWDS PROMs data analysis demonstrated similarly encouraging outcomes for cKP patients, as previously reported for several other body regions. Knee injury and degeneration subgroups had near-equivalent benefits, as observed for all knee conditions. Despite many reported methodological limitations, which limit causal inference and preclude broader recommendations, HWDS appears to potentially offer several benefits for refractory cKP patients, requiring further studies.

## 1. Introduction

Knee pain affects up to 25% of adults, with reported prevalence increasing almost 65% over the past two decades [1,2]. The incidence of chronic knee pain (cKP, 19%) ranks second behind chronic low back pain (cLBP, 23%), just ahead of chronic shoulder pain (cSP, 16%), which collectively results in significant societal disability and healthcare expense [3]. A large Swedish registry study concluded that while there is a need for more relevant evidence-based clinical guidelines, and even though LBP was the most common diagnosis, knee osteoarthritis accounted for the highest number of visits and utilization of physiotherapy resources [4]. The primary pain generators in the knee are complex, with receptors being identified within synovium, ligaments, capsule, subchondral bone, and surrounding soft tissues, but not in articular cartilage; regulation occurs at the spinal and cortical level, influenced by psychosocial factors [5].

While cKP is often initiated with injury/trauma, arthritis is the primary cause in those over 50 years of age, where prosthetic surgery may eventually be required [6,7,8,9]. Medications including narcotics, corticosteroids, non-steroidal anti-inflammatory drugs, and acetaminophen are often used, in combination with multiple non-surgical treatments like physical therapy, intra-articular injections (steroidal and hyaluronic acid), radiofrequency or cryoneurolysis nerve ablation, and possibly regenerative medicine alternatives (platelet-rich plasma and mesenchymal stem cells) [6,7,8,9,10,11,12]. Corticosteroids and opioids may provide temporary relief, but have problematic side effects, failing to target specific pain generators [13,14,15,16,17].

Electrical stimulation (ES) alternatives for knee pain include transcutaneous electrical nerve stimulation (TENS), H-Wave device stimulation (HWDS), interferential current (IFC) or therapy (IFT), pulsed electromagnetic field therapy (PEMF), and neuromuscular electrical stimulation (NMES) [13,14,15]. A meta-analysis of 27 clinical trials, including six forms of ES (HWDS not included)—citing significant methodological problems of heterogeneity and small sample sizes—suggested that IFC might offer some benefits for knee osteoarthritis [18]. Another systematic review/meta-analysis of TENS for knee osteoarthritis suggested that active TENS resulted in slightly greater pain relief than sham TENS, and that it might enhance other interventions (e.g., physical therapy), although it was ineffective for pain associated with stiffness [19]. In contrast, a high-quality Swiss multicenter randomized clinical trial (n = 220) demonstrated no difference in outcomes or benefits between TENS and placebo TENS for knee osteoarthritis [20]. This reconfirms several Cochrane systematic reviews that continue to be unable to confirm TENS’ effectiveness for knee osteoarthritis or general pain conditions [21,22]. This is in accordance with another more recent review, which reported no additional benefits in pain relief or function from TENS compared to sham treatment coupled with exercise and education programs, where a significant placebo effect was noted [23]. TENS for other body areas has shown only short-lived marginal improvement in reported pain, 0.884/10 on the Visual Analog Scale, with no related improvement in quality of life (QoL) or function measures [13,14,15,24,25]. One review highlighted only general short-term pain relief with transcranial direct current stimulation [26]. A recent meta-analysis reported inadequate evidence for NMES coupled with exercise for management of knee osteoarthritis in terms of patient-reported outcome measures (PROMs) [27].

The H-Wave^®^ device has been cleared by the FDA for 15 specific indications, under four classifications, including chronic, post-surgical, acute, and temporary pain; relaxation of muscle spasm, prevention or retardation of disuse atrophy, increasing local blood circulation, muscle re-education, immediate post-surgical stimulation of calf muscles to prevent venous thrombosis, maintaining or increasing range of motion; anesthesia in general dentistry; and muscle spasms associated with temporomandibular joint (TMJ) [13,14,15].

Numerous H-Wave^®^ studies have reported significant improvements in function, pain, sleep, and QoL [13,14,15]. HWDS uses a unique proprietary, biphasic exponentially decaying waveform (0–35 mA current, 0–35 V voltage at 1000 ohms load, ultra-long 5000-microsecond pulse duration), having low-frequency (2 Hz) and high-frequency (60 Hz) modes [13,14,15]. Multiple mechanisms of action of both modes have been described in detail in related HWDS publications, which involve physiological soft tissue benefits and potent analgesic effects [13,14,15]. Further HWDS studies are warranted since reported clinical outcomes have been more robust than for other forms of ES.

Specialty societies have encouraged studies involving PROMs, which are increasingly respected for evidence-based quality. Patient-reported outcomes (PROs) reflect honest assessments of patient health conditions [28]. The National Quality Forum (NQF) states that “PROs can be any description of the patient’s condition, behavior, or experience with treatment that arises straight from the patient, without interpretation of the patient’s answer by a clinician or anyone else” [28]. PROMs specify the “instruments, metrics, or tools utilized (e.g., scales, single-item measures) for evaluation of patient-reported health status” [29].

Previously published PROMs studies on H-Wave^®^ efficacy for neck, shoulder, and non-specific chronic low back pain reported equivalent positive outcomes, regardless of body area [13,14,15]. This was not surprising, since the initially reported cLBP outcomes (n = 2711) turned out to be notably similar to those for “all diagnoses” (n = 11,503) [13]. We now hypothesize that HWDS outcomes for cKP will also be similarly encouraging, with the goal of demonstrating significant benefits for pain, function, sleep quality, and drug cessation, with secondary objectives to perform subgroup analyses in patients with knee injury versus degeneration.

## 2. Materials and Methods

### 2.1. Data Source and Study Design

As more fully described in previous publications, the manufacturer of H-Wave^®^ (Electronic Waveform Lab, Inc., Huntington Beach, CA, USA) routinely and consecutively collects proprietary outcome survey data from user patients, consisting of validated pain and single-item (valid by definition) PROMs. The rationale for the study design is that it takes advantage of large sample sizes and direct patient-reported outcomes, despite various methodological limitations. Over a 4-year period (2019–2022), 34,192 pain management patients returned surveys, where only the most recently submitted ones per patient were analyzed for this study’s purposes. The prospective data was retrospectively evaluated for statistical significance, independently, externally, and without interference, with Electronic Waveform Lab, Inc. allowing the analysis of the untampered raw dataset [13,14,15]. The institutional review board of South Texas Orthopaedic Research Institute, according to the Declaration of Helsinki, approved this study (STORI051624-1, 1 October 2024). The informed consent to gather data and assess it for publication was prospectively obtained from the participating patients. No protected health information (PHI) was analyzed or reported. Electronic Waveform Lab, Inc. gave consent for this entire raw dataset to be independently reviewed, without interference.

This study strictly targets H-Wave^®^ device use for any identified knee disorder in patients 18 years and older, with pain chronicity prior to HWDS initiation from 90 to 730 days (3–24 months), with device treatment duration from 22 to 365 days. Diagnoses included ankylosis, left/right knee; bilateral primary osteoarthritis of knee; bucket-handle tear of medial meniscus; chondromalacia; chondromalacia patellae; chronic instability of knee; complex tear of lateral/medial meniscus, current injury; contusion of left/right knee; derangement of posterior horn of medial meniscus, old tear/injury; osteoarthritis of knee; other bursitis of knee; other instability, left/right knee; other internal derangements of left/right knee; other meniscus derangement; other specified joint disorders, left/right knee; other spontaneous disruption of anterior cruciate ligament (ACL); other spontaneous disruption of medial collateral ligament (MCL); other tear of lateral/medial/unspecified meniscus; pain in left/right/unspecified knee; patellar tendinitis; patellofemoral disorders; plica syndrome; presence of artificial knee joint; peripheral tear of lateral/medial meniscus; sprain of ACL; sprain of lateral/medial collateral ligament (LCL/MCL); sprain of other specified parts of left/right knee; sprain of posterior cruciate ligament (PCL); sprain of unspecified collateral/cruciate ligament; sprain of unspecified site; stiffness of left/right knee; strain of left/right quadriceps muscle, fascia, and tendon; tear of articular cartilage; traumatic arthropathy, left/right knee; unilateral post-traumatic osteoarthritis; unilateral primary osteoarthritis, left/right knee; unspecified internal derangement of left/right knee; and unspecified tear of unspecified meniscus. All non-knee-related diagnoses were excluded.

For the first knee injury subgroup analysis, the inclusion criteria included patients with bucket-handle tear of medial meniscus; chronic instability of knee; complex tear of lateral/medial meniscus, current injury; derangement of posterior horn of medial meniscus, old tear/injury; other instability, left/right knee; other meniscus derangement; other spontaneous disruption of ACL; other spontaneous disruption of MCL; other tear of lateral/medial/unspecified meniscus; pain in left/right/unspecified knee; peripheral tear of lateral/medial meniscus; sprain of ACL; sprain of LCL/MCL; sprain of other specified parts of left/right knee; sprain of PCL; sprain of unspecified collateral ligament; sprain of unspecified cruciate ligament; sprain of unspecified site; strain of left/right quadriceps muscle, fascia, and tendon; tear of articular cartilage; traumatic arthropathy, left/right knee; unilateral post-traumatic osteoarthritis; and unspecified tear of unspecified meniscus. For the second knee degeneration subgroup analysis, inclusion criteria included patients with ankylosis, left/right knee; bilateral primary osteoarthritis of knee; osteoarthritis of knee; presence of artificial knee joint; stiffness of left/right knee; and unilateral primary osteoarthritis, left/right knee. The exclusion criteria for both subgroups were identical, including all non-knee-related diagnoses.

All participants received consistent and standardized training for proper H-Wave^®^ device home use. Primary study outcomes include H-Wave^®^ effects on self-reported pain, medication usage, function/activities of daily living (ADL), and sleep. Secondary outcomes include work performance, patient satisfaction, and device preference compared to prior treatment. A checklist of STROBE Epidemiology Reporting (for observational studies) was strictly followed.

### 2.2. Data Collection

The participants answered specific questions (Figure S1) regarding their experiences with HWDS, specifically related to effects on function/ADL, pain, medication usage, work, sleep, prior treatments, and device satisfaction, obtained using a previously published questionnaire [13,14,15]. Additionally, gender, age (when injured), pain duration, and specifics of device use were queried. The survey instrument, while proprietary, included several validated PROMs, like Numeric Rating Scale (NRS) pain measures, and single-item questions derived from general (not knee-specific) function outcome instruments, including the Oswestry Disability Index. Many patients completed several survey questionnaires during their treatment, so to avoid duplication and provide consistency, only the final completed survey per participant was analyzed. Also, missing or repeated survey responses were excluded from the data analysis.

### 2.3. Statistical Analysis

Consistent with previous related studies, the independent data analyses consisted of an initial univariate distributional, correlation/association, and contingency table, applying several logistic and linear regression methods on all participant covariates. A stepwise model selection technique resulted in a statistically significant (≤5% significance) and parsimonious model. The statistical programming language R, along with SAS JMP, was employed for data pre-processing and analysis. While linear regressions were performed with stepwise model selection techniques, diagnostics and assessments were completed to check the normality assumption of the residuals [13,14,15].

An a priori power calculation was not performed, justified by the study’s design as a retrospective, descriptive analysis of an existing, large, consecutively collected PROMs database. The objective was to report real-world outcomes to provide robust preliminary evidence for the planning of future, adequately powered, prospective randomized controlled trials.

## 3. Results

### 3.1. Cohort and Inclusion/Exclusion Criteria

**Figure 1:**
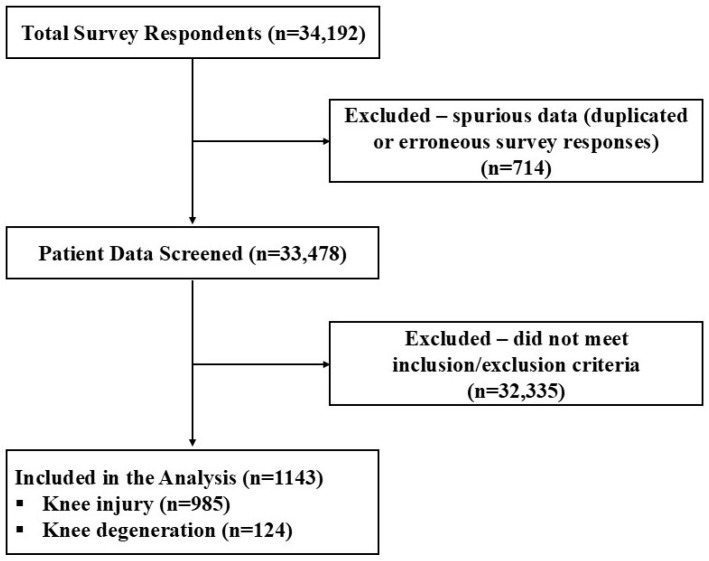
Inclusion/exclusion flow diagram.

Out of 34,192, 1143 knee participants who fulfilled the inclusion criteria were subject to data assessment. Gender distribution was fairly uniform, with a slightly lower proportion of females (47.59%) than males (52.41%) (Figure 1, Table 1). Average age at date of injury and onset of HWDS treatment were 47.96 ± 11.59 and 48.82 ± 11.61 years (±standard deviation), respectively. Average reported pain length and H-Wave^®^ duration of use were 310.85 ± 179.25 and 93.33 ± 65.55 days, respectively (Table 1).

For subgroup analysis, the inclusion criteria were met by 985 knee injury patients (Figure 1). Gender distribution showed a lower proportion of females (46.60%) than males (53.40%). Average age at date of injury and onset of HWDS treatment were 47.66 ± 11.55 and 48.50 ± 11.56 years, respectively. Average reported pain length and H-Wave^®^ duration of use were 306.78 ± 178.09 and 93.15 ± 65.80 days, respectively (Table 1).

Only 124 patients fulfilled the inclusion criteria with knee degeneration (Figure 1). Gender distribution showed a lower proportion of females (41.13%) than males (58.87%). Average age at date of injury and onset of HWDS treatment were 52.42 ± 10.72 and 53.47 ± 10.80 years, respectively. Average reported pain length and H-Wave^®^ duration of use were 380.42 ± 199.22 and 92.80 ± 66.80 days, respectively (Table 1).

In summary, while both subgroups appear to be similar across several demographic characteristics, the knee degeneration group had an older, more predominantly male population and longer pain chronicity compared to the knee injury group, with identical HWDS usage.

### 3.2. Device Usage

Of the 1143 knee patients, 1111 utilized the device twice a day (1.91 ± 0.89), and 1094 used it five and a half days a week (5.67 ± 1.61). Of 1129 patients with available data, over half (52.52%) used it for 30–45-min treatment sessions.

Of the 985 knee injury patients, 958 utilized the device twice a day (1.90 ± 0.89), and 944 used it five and a half days a week (5.66 ± 1.61). Of 974 patients with available data, over half (53.18%) used it for 30–45-min treatment sessions.

Of the 124 patients with knee degeneration, 119 utilized the device twice a day (1.93 ± 0.71), and 118 used it five and a half days a week (5.64 ± 1.71). Of 122 patients with available data, over half (53.28%) used it for sessions lasting 30 to 45 min.

### 3.3. Insurance Mix

This knee investigation cohort mostly comprised workers’ compensation claimants (n = 1021, 89.48%). Other claimants included personal injury (n = 86, 7.54%), auto injury (n = 32, 2.81%), and Tricare patients (n = 2, 0.17%).

A similar mix was observed in knee injury patients, with 88.71% workers’ compensation (n = 872), 8.34% personal injury (n = 82), 2.75% auto injury (n = 27), and 0.20% Tricare (n = 2) claimants. In contrast, patients with knee degeneration included 98.39% workers’ compensation (n = 122) and 1.61% auto injury claimants (n = 2).

### 3.4. Concomitant Home Exercise Program

Of 1100 patients with available data, over two-thirds (n = 776, 70.55%) participated in home exercise, with the others (n = 324, 29.45%) not doing so. Of 947 patients with knee injury, over two-thirds (n = 668, 70.54%) participated in home exercise, with the others (n = 279, 29.46%) not doing so. Of 120 patients with knee degeneration, two-thirds (n = 80, 66.67%) participated in home exercise, with the others (n = 40, 33.33%) not doing so.

### 3.5. Primary Outcome Measures

#### 3.5.1. Pain Reduction

**Figure 2:**
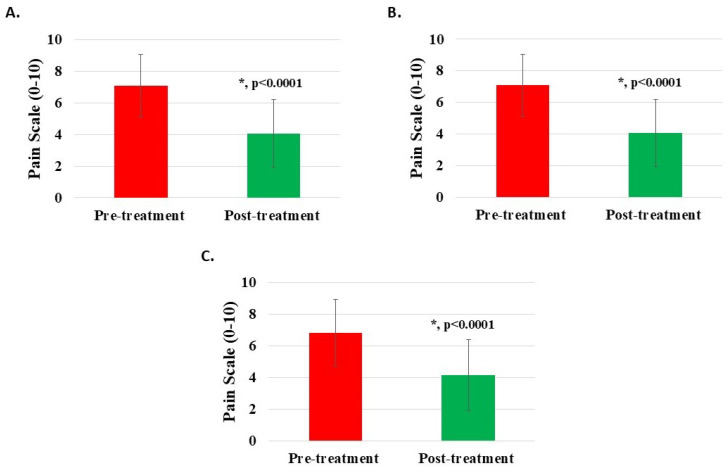
Pain reduction post-treatment with H-Wave^®^ device stimulation for (**A**) all knee disorders, (**B**) knee injury, and (**C**) knee degeneration. A difference of 2 or more points was statistically significant for all groups (*, *p* < 0.0001).

The pre-treatment Numeric Rating Scale (NRS) for 1130 knee patients averaged 7.06 ± 1.98 (95% confidence interval: 6.94, 7.17), while post-treatment (for 1127) averaged 4.07 ± 2.14 (95% confidence interval: 3.94, 4.20). The improvement difference between post-treatment and pre-treatment (for 1125) was 2.96 ± 1.77 (95% confidence interval: 2.86, 3.06). An NRS difference of 2 or more was statistically significant (*p* < 0.0001) (Figure 2A, Table 2). Half (n = 257, 50.59%) of the patients with NRS ratings of 8 or higher (n = 508, 44.96%) before treatment reduced to 5 or less after treatment.

For subgroup analysis, the pre-treatment NRS for 974 patients with knee injury averaged 7.09 ± 1.95 (95% confidence interval: 6.97, 7.21), while post-treatment (for 972) averaged 4.07 ± 2.12 (95% confidence interval: 3.94, 4.21). The improvement difference between post-treatment and pre-treatment (for 971) was 2.99 ± 1.76 (95% confidence interval: 2.88, 3.10). An NRS difference of 2 or more was statistically significant (*p* < 0.0001) (Figure 2B, Table 2). Half (n = 223, 51.15%) of the knee injury patients with NRS ratings of 8 or higher before treatment reduced to 5 or less following treatment. Likewise, the pre-treatment NRS for 123 patients with knee degeneration averaged 6.85 ± 2.11 (95% confidence interval: 6.47, 7.22), while post-treatment (for 122) averaged 4.14 ± 2.25 (95% confidence interval: 3.74, 4.54). The improvement difference between post-treatment and pre-treatment (for 122) was 2.72 ± 1.74 (95% confidence interval: 2.41, 3.03). An NRS difference of 2 or more was statistically significant (*p* < 0.0001) (Figure 2C, Table 2). Only 22 patients (41.51%) with knee degeneration with NRS ratings of 8 or higher before treatment reduced to 5 or less following treatment.

**Figure 3:**
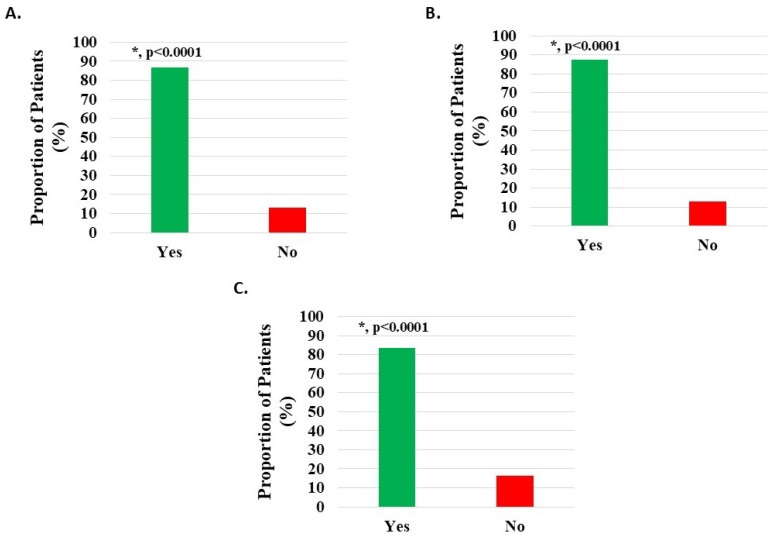
Pain relief of at least 20% post-treatment with H-Wave^®^ device stimulation for (**A**) all knee disorders, (**B**) knee injury, and (**C**) knee degeneration. This was statistically significant for all groups (*, *p* < 0.0001).

Pain reduction of 20% or more has been previously considered as a reasonable estimate for the minimal clinically important difference (MCID) for H-Wave^®^ [15]. Of 1125 knee subjects, 976 (86.76%) documented post-treatment pain relief of 20% or more (*p* < 0.0001) (Figure 3A). Of 971 patients with knee injury, 847 (87.23%) documented post-treatment pain relief of 20% or more (*p* < 0.0001) (Figure 3B). Of 122 patients with knee degeneration, 102 (83.61%) documented post-treatment pain relief of 20% or more (*p* < 0.0001) (Figure 3C).

#### 3.5.2. Function/ADL Improvement

**Figure 4:**
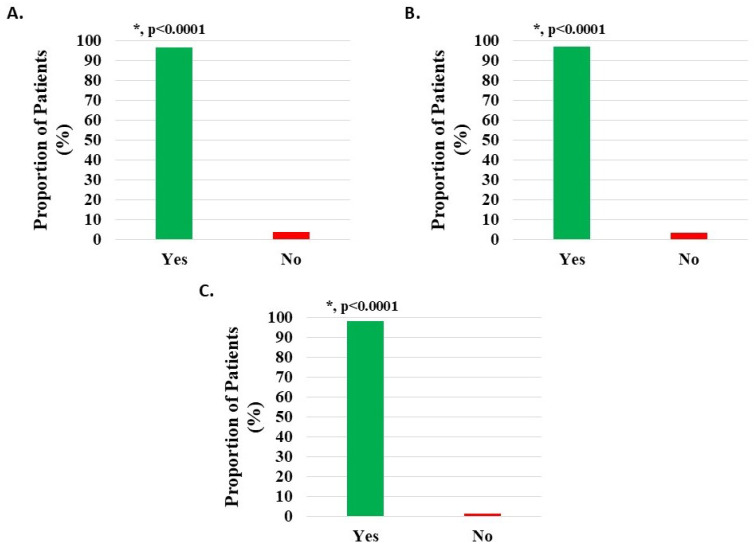
Improvement in function/ADL post-treatment with H-Wave^®^ device stimulation for (**A**) all knee disorders, (**B**) knee injury, and (**C**) knee degeneration. The improvement was statistically significant for all groups (*, *p* < 0.0001).

Of 1090 HWDS knee subjects (excluding 53 with missing survey data, Table S1), 1052 (96.51%) documented statistically significant improvement (*p* < 0.0001) in post-treatment function (Figure 4A, Table 2).

Of 938 knee injury subjects (excluding 40 with missing data), 907 (96.70%) documented statistically significant improvement (*p* < 0.0001) in post-treatment function (Figure 4B, Table 2).

Of 120 knee degeneration subjects (excluding 4 with missing data), 118 (98.33%) documented statistically significant improvement (*p* < 0.0001) in post-treatment function (Figure 4C, Table 2).

#### 3.5.3. Medication Usage Decrease

**Figure 5:**
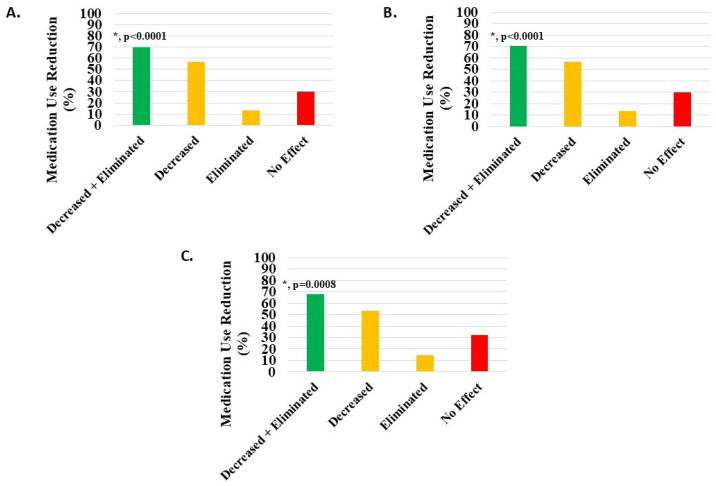
Decrease or elimination in use of pain medications post-treatment with H-Wave^®^ device stimulation for (**A**) all knee disorders, (**B**) knee injury, and (**C**) knee degeneration. Decreased + eliminated versus no effect was statistically significant for all groups (*, *p* < 0.0001, *p* < 0.0001, and *p* = 0.0008, respectively).

Of 806 knee patients (excluding 337 with missing data), 70% (n = 563, 69.85%) reported a statistically significant (*p* = 0.0008) reduction or cessation of pain medication use. Post-treatment, 457 patients (56.70%) reduced and 106 (13.15%) eliminated pain medications (Figure 5A, Table 2).

Of 694 knee injury patients (excluding 291 with missing data), 488 (70.32%) reported a statistically significant (*p* < 0.0001) reduction or cessation of pain medication use. Post-treatment, 393 patients (56.63%) reduced and 95 (13.69%) eliminated pain medications (Figure 5B, Table 2).

Of 81 knee degeneration patients (*excluding 43 with missing data*), 55 (67.90%) reported a statistically significant (*p* = 0.0008) reduction or cessation of pain medication use. Post-treatment, 43 patients (53.09%) reduced and 12 (14.81%) eliminated pain medications (Figure 5C, Table 2).

#### 3.5.4. Sleep Improvement

**Figure 6:**
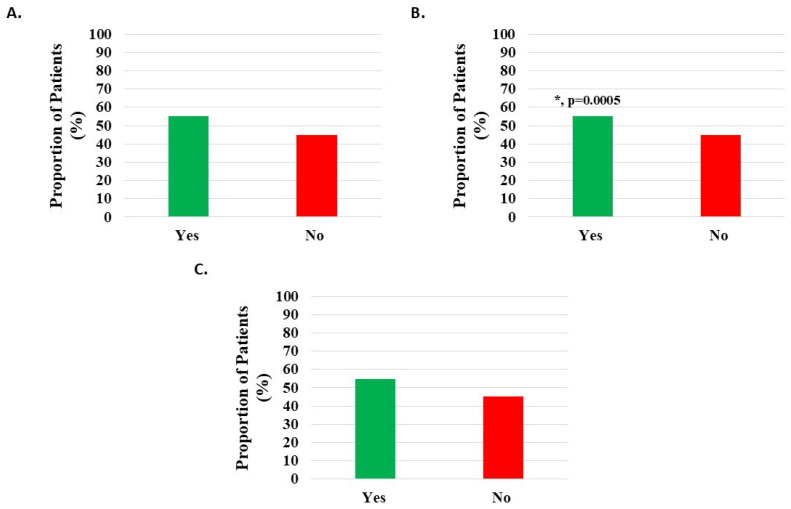
Improvement in quality of sleep post-treatment with H-Wave^®^ device stimulation for (**A**) all knee disorders, (**B**) knee injury, and (**C**) knee degeneration. The improvement was statistically significant for only the knee injury group (*, *p* = 0.0005).

Of 1143 knee subjects, 630 (55.12%) indicated sleep quality improvement; however, this difference was not statistically significant (Figure 6A, Table 2).

Of 985 patients with knee injury, 545 (55.33%) indicated statistically significant (*p* = 0.0005) sleep quality improvement (Figure 6B, Table 2).

Of 124 patients with knee degeneration, 68 (54.84%) indicated sleep quality improvement; however, the difference was not statistically significant (Figure 6C, Table 2).

### 3.6. Secondary Outcome Measures

#### 3.6.1. Work Status and Performance

**Figure 7:**
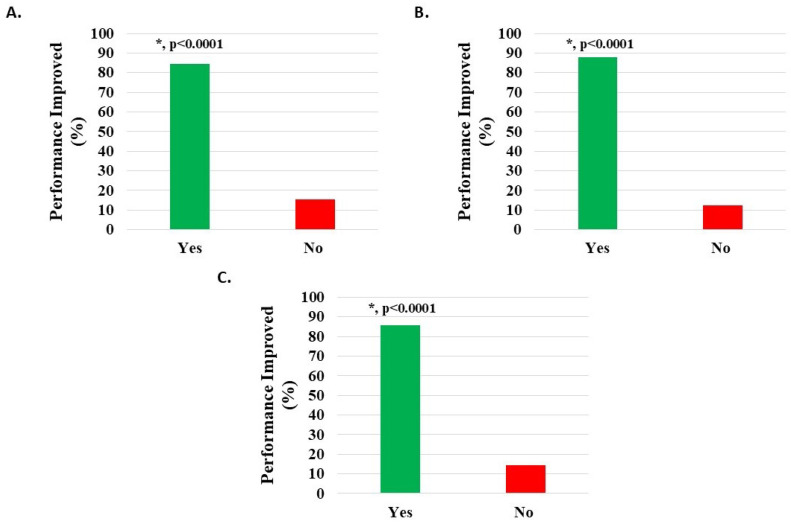
Improvement in work performance in patients on full or modified duty post-treatment with H-Wave^®^ device stimulation for (**A**) all knee disorders, (**B**) knee injury, and (**C**) knee degeneration. The improvement was statistically significant for all groups (*, *p* < 0.0001).

Of 1083 knee subjects (excluding 60 with missing data), 634 (58.54%) were off work when starting H-Wave^®^, while 235 (21.70%) performed modified work, and 214 (19.76%) were in full-time work. Of the 634 subjects off work, 339 related this to their injury, and of these, 143 (42.18%) indicated that H-Wave^®^ had assisted their return to work. Data analysis from those not working suggested that the larger the difference between reported pain levels before and after HWDS, the more likely HWDS was to help them return to work (*p* < 0.0001, 95% confidence interval: 0.274, 0.607). Of 423 subjects (excluding 26 with missing survey data) performing full or modified work, 358 (84.63%) reported statistically significant (*p* < 0.0001) improvement in performance at work following treatment with H-Wave^®^ (Figure 7A, Table 2). Additional analysis suggested that H-Wave^®^ is more likely to improve performance at work for those whose pain was reduced by at least 20% after treatment (odds ratio = 25.215; 95% confidence interval: 11.461, 57.888).

Of 935 knee injury subjects (excluding 50 with missing data), 547 (58.50%) were off work when starting H-Wave^®^, while 205 (21.93%) performed modified work, and 183 (19.57%) were in full-time work. Of the 547 subjects off work, 289 related this to their injury, and of these, 128 (44.29%) indicated that H-Wave^®^ had assisted their return to work. Data analysis from those not working suggested that the larger the difference between pain levels before and after HWDS, the more likely HWDS was to help them return to work (*p* < 0.0001, 95% confidence interval: 0.227, 0.572). Of 359 subjects (excluding 29 with missing survey data) performing full or modified work, 315 (87.74%) reported statistically significant (*p* < 0.0001) improvement in performance at work following treatment with H-Wave^®^ (Figure 7B, Table 2). Additional analysis suggested that H-Wave^®^ is more likely to improve performance at work for those whose pain was reduced by at least 20% after treatment (odds ratio = 22.117; 95% confidence interval: 8.516, 59.712).

Of 119 knee degeneration subjects (excluding 5 with missing data), 74 (62.18%) were off work when starting H-Wave^®^, while 19 (15.97%) performed modified work, and 26 (21.85%) were in full-time work. Of the 74 subjects off work, 37 related this to their injury, and of these 37, 14 (37.84%) indicated that H-Wave^®^ had assisted their return to work. Of 42 subjects (excluding 3 with missing survey data) performing full or modified work, 36 (85.71%) reported statistically significant (*p* < 0.0001) improvement in performance at work following treatment with H-Wave^®^ (Figure 7C, Table 2). Additional analysis suggested that H-Wave^®^ is more likely to improve performance at work for those whose pain was reduced by at least 20% after treatment (odds ratio = 61.999; 95% confidence interval: 6.077, 1624.832).

#### 3.6.2. Prior Treatment and Preference for HWDS

Of 1143 knee subjects, 1121 (98.07%) used other treatment modalities prior to H-Wave^®^ (*p* < 0.0001). Of 1092 patients (excluding 51 with missing data), 743 (68.04%) reported that H-Wave^®^ was significantly (*p* < 0.0001) more helpful than prior treatments, while 328 (30.04%) noted similar efficacy, and 21 (1.92%) less efficacy (Table 2).

Of 985 knee injury subjects, 967 (98.17%) used other treatment modalities prior to H-Wave^®^ (*p* < 0.0001). Of 944 patients (excluding 41 with missing data), 654 (69.28%) reported that H-Wave^®^ was significantly (*p* < 0.0001) more helpful than prior treatments, while 277 (29.34%) noted similar efficacy, and 13 (1.38%) less efficacy (Table 2).

Of 124 knee degeneration subjects, 123 (99.19%) used other treatment modalities prior to H-Wave^®^ (*p* < 0.0001). Of 118 patients (excluding 6 with missing data), 78 (66.10%) reported that H-Wave^®^ was significantly (*p* < 0.0001) more helpful than prior treatments, while 36 (30.51%) noted similar efficacy, and 4 (3.39%) less efficacy (Table 2).

#### 3.6.3. Patient Expectations

**Figure 8:**
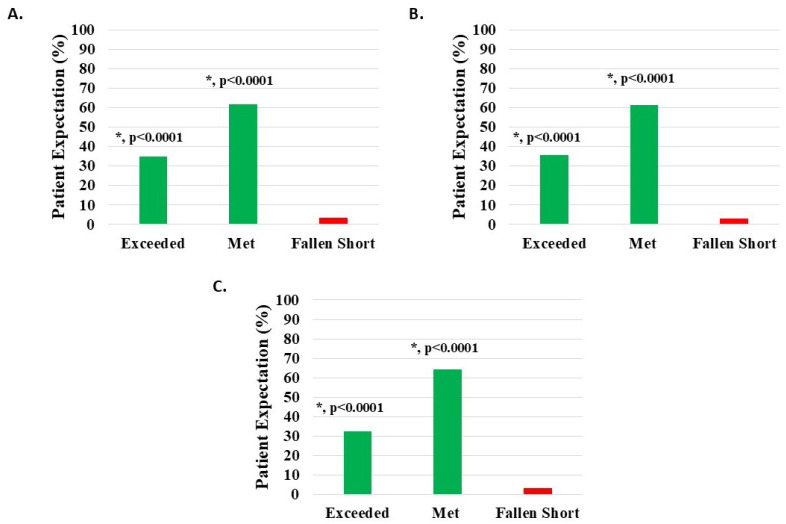
Level of patient expectations, where the proportion reporting H-Wave^®^ device stimulation either exceeded or met expectations, was statistically significant (*, *p* < 0.0001) for all groups analyzed: (**A**) all knee disorders, (**B**) knee injury, and (**C**) knee degeneration.

Of 1094 knee subjects (excluding 49 with missing data), 1056 (96.53%) reported that HWDS met or exceeded expectations; 674 (61.61%) noted met expectations and 382 (34.92%) exceeded expectations, both statistically significant (*p* < 0.0001). Expectations were not met in 38 patients (3.47%) (Figure 8A, Table 2).

Of 942 knee injury patients (excluding 43 with missing data), 912 (96.82%) reported that HWDS met or exceeded expectations; 578 (61.36%) noted met expectations and 334 (35.46%) exceeded expectations, both statistically significant (*p* < 0.0001). Expectations were not met in 30 patients (3.18%) (Figure 8B, Table 2).

Of 120 knee degeneration patients (excluding 4 with missing data), 116 (96.67%) reported that HWDS met or exceeded expectations; 77 (64.17%) noted met expectations and 39 (32.50%) exceeded expectations, both statistically significant (*p* < 0.0001). Expectations were not met in four patients (3.33%) (Figure 8C, Table 2).

#### 3.6.4. Patient Satisfaction with Service

**Figure 9:**
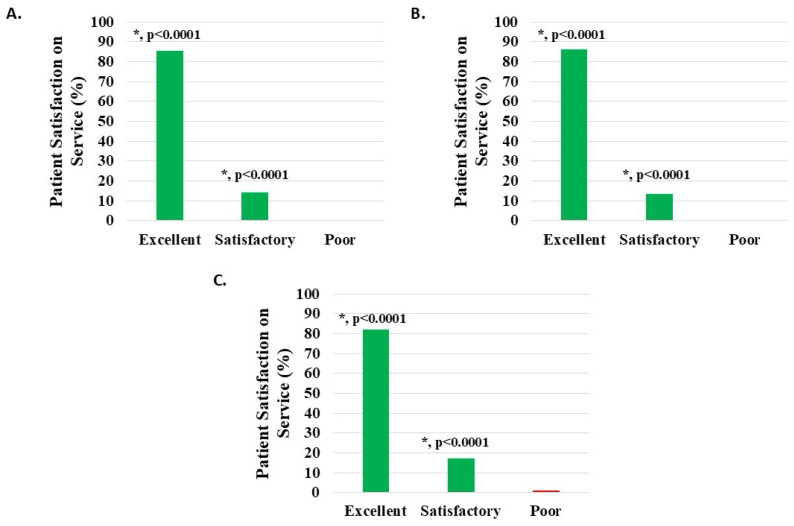
Level of patient satisfaction with service, where the proportion reporting H-Wave^®^ device stimulation instruction to be excellent or satisfactory was statistically significant (*, *p* < 0.0001), for all groups analyzed: (**A**) all knee disorders, (**B**) knee injury, and (**C**) knee degeneration.

Of 1114 knee subjects (excluding 29 with missing data), 1109 (99.55%) reported that the H-Wave^®^ team service was satisfactory or excellent; 158 (14.18%) noted satisfactory and 951 (85.37%) excellent, both statistically significant (*p* < 0.0001). Poor service was noted in five patients (0.45%) (Figure 9A, Table 2).

Of 958 knee injury subjects (excluding 27 with missing data), 955 (99.69%) reported that the H-Wave^®^ team service was satisfactory or excellent; 130 (13.57%) noted satisfactory and 825 (86.12%) excellent, both statistically significant (*p* < 0.0001). Poor service was noted in three patients (0.31%) (Figure 9B, Table 2).

Of 122 knee degeneration subjects (excluding 2 with missing data), 121 (99.18%) reported that the H-Wave^®^ team service was satisfactory or excellent; 21 (17.21%) noted satisfactory and 100 (81.97%) excellent, both statistically significant (*p* < 0.0001). Poor service was noted in one patient (0.82%) (Figure 9C, Table 2).

#### 3.6.5. Patient Confidence in Device Use

**Figure 10:**
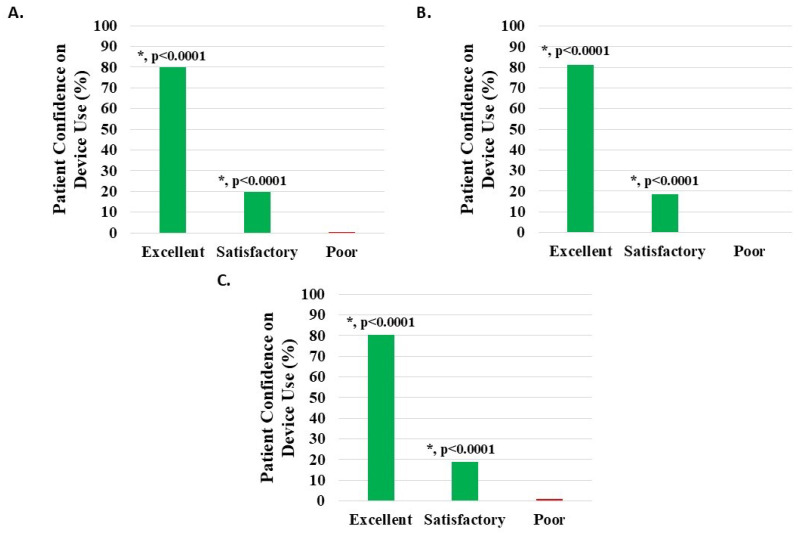
Level of patient confidence in device use, where the proportion reporting H-Wave^®^ device stimulation use on their own being excellent or satisfactory was statistically significant (*, *p* < 0.0001) for all groups analyzed: (**A**) all knee disorders, (**B**) knee injury, and (**C**) knee degeneration.

Of 1112 knee subjects (excluding 31 with missing data), 1108 (99.64%) reported device usage confidence to be satisfactory or excellent; 218 (19.60%) noted satisfactory and 890 (80.04%) excellent, both statistically significant (*p* < 0.0001). Poor confidence was noted in four patients (0.36%) (Figure 10A, Table 2).

Of 956 knee injury subjects (excluding 29 with missing data), 953 (99.69%) reported device usage confidence to be satisfactory or excellent; 178 (18.62%) noted satisfactory and 775 (81.07%) excellent, both statistically significant (*p* < 0.0001). Poor confidence was noted in three patients (0.31%) (Figure 10B, Table 2).

Of 122 knee degeneration subjects (excluding 4 with missing data), 121 (99.18%) reported device usage confidence to be satisfactory or excellent; 23 (18.85%) noted satisfactory and 98 (80.33%) excellent, both statistically significant (*p* < 0.0001). Poor confidence was noted in one patient (0.82%) (Figure 10C, Table 2).

### 3.7. Outcomes Based on Treatment Period

Three subgroups were stratified by number of days of H-Wave^®^ use, including a “trial period” from 22 to 35 days (3 to 5 weeks), an “early treatment period” from 36 to 98 days (5 to 14 weeks), and a “late treatment period” from 99 to 365 days (14 to 52 weeks); sample sizes were 320 (28%), 299 (26.16%), and 524 (45.84%) knee patients, respectively. Outcomes were generally consistent regardless of treatment duration, although device use for longer periods correlated with more pain relief, medication cessation, improved sleep, and better work performance (Table 3).

Additional findings were observed through supplementary multiple regression analyses of statistical significance, where duration of both device use and pain chronicity were important variables affecting the outcomes. Statistically significant inferences include the following:

- Device usage over longer periods resulted in greater pain reduction (*p* < 0.0001);
- Less pain reduction occurred with longer chronicity of pain (*p* < 0.0001);
- The greater the difference in reported pain level before and after treatment, the more likely HWDS improved sleep (*p* < 0.0001);
- Function/ADL improved more when pain levels were reduced by at least 20% following treatment (*p* < 0.0001).

### 3.8. Outcomes Based on Longer Pain Chronicity

The outcomes reported above represent the study restriction of pain chronicity between 3 and 24 months, but an analysis was also performed, including more chronic patients up to 36 months. Sample size for “all knee diagnoses” increased from 1143 to 1352 patients. Outcomes were equivalent for improvements in function/ADL (96.51% at 2 years, 96.35% at 3 years), work performance (84.63% at 2 years, 84.92% at 3 years), and medication reduction (69.85% at 2 years, 68.42% at 3 years). While other results remained positive after adding the additional patients, slightly less robust outcomes were observed in average reported pain level reduction (2.96% at 2 years, 2.55% at 3 years), return-to-work (42.18% at 2 years, 31.07% at 3 years), and sleep improvement (55.12% at 2 years, 48.99% at 3 years).

## 4. Discussion

This is a PROMs study of the potential effectiveness of HWDS for treatment of chronic knee pain conditions. Surveys meeting the study criteria included those from 1143 “all knee diagnoses” patients, with subgroup analyses performed for 985 knee injury and 124 degenerative knee conditions (e.g., osteoarthritis, stiffness, and joint replacement). As previously and consistently demonstrated for low back, neck, and shoulder conditions—primarily in injured workers [13,14,15]—clinical outcomes were positive and encouraging. Given the magnitude of reported improvements in function, pain, sleep, and decreased medication use, HWDS appears to be a reasonable consideration for otherwise difficult-to-treat refractory painful knee conditions resulting from injury or degeneration.

The most common site of musculoskeletal pain is the back, followed by the knee, which accounts for almost 4 million visits annually to primary care providers (many more directly to specialists), where physical disability rises with age and becomes more symptomatic in areas of higher social deprivation [1,2,3]. Osteoarthritis (OA) and related degenerative conditions are progressive by nature, accounting for significant disability, with a highly variable incidence of pain and a poor correlation to imaging; treatment is not definitive, as surgery often fails to guarantee improvement [2,3]. Physical therapy, corticosteroid injection, and viscosupplementation have been most widely studied for non-surgical knee treatment, while nerve ablation (thermal, radiofrequency, and cryoneurolysis) and geniculate artery synovial embolization are alternatives currently under study [12]. Intra-articular injections have demonstrated mild effects lasting limited periods of time, prompting research in regenerative medicine—particularly platelet-rich plasma or mesenchymal stem cells—in the hopes of achieving more durable pain relief [6,7,8,9,10,11].

Electrical stimulation (ES) has gained interest as an alternative non-surgical, non-invasive management modality, either used stand-alone or as an adjunct. Some authors suggest that interferential current (IFC) might offer the most promise for knee OA, while others more recently have reported robust improvements in general function and chronic pain with HWDS [13,14,15,18]. In contrast, TENS, the most prescribed form of ES, has been demonstrated to be relatively ineffective for knee OA pain, despite promising pre-clinical studies [20,30]. Systematic reviews and meta-analyses comparing different forms of ES for knee pain remain limited, with almost no direct comparisons between devices, limiting definitive guidelines support [18,20,21]. As an example, Clinical Practice Guidelines for non-surgical treatment of knee OA by the American Academy of Orthopaedic Surgeons only offer limited recommendations for TENS, PEMF, and percutaneous electrical nerve stimulation [31].

PROMs are being increasingly reported as a measure of treatment effectiveness, efficiency, and patient satisfaction, providing essential outcomes from the patient’s perspective, supporting value-based healthcare models [32]. As observed in the previously published shoulder PROMs study, HWDS for the knee appears to be similarly effective, not only to positive shoulder outcomes, but also to those reported for chronic spinal pain [13,14,15]. Differences in treatment effects between knee injury and degenerative (primarily OA) conditions were minimal. Pain chronicity up to 24 months was reported, consistent with earlier publications on low back and neck, but data was additionally analyzed for up to 36 months (increasing sample size), with only minor notable differences in statistical outcomes. Workers’ compensation claimants dominated the study cohort—patients who generally have poorer treatment outcomes—but even so, knee outcomes were similarly compelling, consistent with multiple previous HWDS studies.

Here, knee pain reduction with HWDS was almost 3 (2.96) on a scale of 10, significantly higher than TENS (0.88/10) for generalized conditions [13,14,15]. Specifically, pain reduction of 20% or more (MCID) was reported by 87% of knee patients. Improvements in function were self-reported in almost 97%, with work performance in those working improving in almost 85%. Almost 70% of patients on medications decreased or stopped them, with sleep improvement reported by 55% of knee injury participants. The lack of statistical significance for sleep improvement in the knee degeneration group can be attributed to the limited sample size, instead of an absence of true therapeutic effect. Patient satisfaction was 97% or higher for expectations, service, and confidence with the device. Moreover, an analysis including additional patients with pain chronicity beyond two and up to three years found equivalent outcomes for function/ADL, work performance, and medication reduction, while slightly lower average pain level reduction, return-to-work times, and sleep improvements were noted. This is similar to prior low back, neck, shoulder, and other HWDS publications, where it has been repeatedly demonstrated that longer duration of pain leads to a lower reduction in reported pain [13,14,15].

Adverse events (AEs), either mild or severe, were not specifically analyzed in this database, even though patients could report them under comments (question #13, Figure S1). The manufacturer methodically collects AE occurrences separately from the survey, mostly consisting of minor skin irritation beneath the adhesive lead pads. Prior non-PROMs H-Wave studies have consistently reported rare and benign safety issues, typical of FDA-approved Class II devices [13,14,15].

### Limitations and Future Directions

The authors fully acknowledge that this is a single-center, observational, retrospective analysis of a convenience sample, primarily workers’ compensation claimants, necessitating a guarded generalization of the findings. A priori sample size calculation was not performed due to the retrospective design. However, the large cohort of over 1000 patients supports generating hypotheses for future studies. The manuscript reports all relevant outcomes and 95% confidence intervals. For the primary pain outcome, we currently report a mean NRS reduction of 2.96 points (95% CI: 2.86, 3.06).

Additional limitations beyond the retrospective design (prospective data collection) and possible risk of selection bias (workers’ compensation cohort) include lack of a control group and inherent risk of bias in the manufacturer-distributed survey (voluntary convenience). No BMI was determined for either group. Concurrent treatments, with the exception of home exercise, were not specifically reported, nor were any co-morbidities, further confounding interpretation. This study also lacks the computation of T-scores. Additionally, this study lacks a formal power calculation and has the potential for residual confounding from non-adjusted descriptive analyses, acknowledging that the one-year maximum treatment duration analyzed constitutes a limitation on long-term follow-up. Inclusion criteria included differing pathophysiologies such as acute injuries, chronic degeneration, post-traumatic osteoarthritis, and post-arthroplasty, with different expected responses.

The proprietary survey instrument was partially unvalidated, although it included valid NRS and multiple single-item questions. Specific instruments such as KOOS, WOMAC, and the Oxford Knee Score would be more appropriate for future condition-specific studies. The final survey was defined as only the final completed survey per participant to provide consistency and prevent patient duplication, although this approach could be a source of inherent survivorship and responder bias. Study improvements might have included data collected from individual participants over time and more specific safety reporting.

Multivariate analyses were performed using stepwise linear and logistic regression models to identify parsimonious models from participant covariates. While these models attempt to account for some variables, the reliance on descriptive statistics for primary outcomes and the retrospective nature of this study mean that potential confounders, such as concurrent treatments (other than recorded home exercise), were not completely controlled for, where the lack of comprehensive adjustment could potentially affect the interpretability, warranting the recommendation of guarded generalization of the results.

The current data format and absence of certain auxiliary information prevented the quantification of response rates relative to device distribution, a comparison between responders and non-responders, and a comprehensive sensitivity analysis comparing early versus final surveys. Regarding a substantial proportion of missing data, such data is neither missing at random (MAR) nor missing completely at random (MCAR), suggesting systematic missingness, requiring the use of a transparent complete case analysis by excluding cases with missing values during subgroup analysis, acknowledging that this reduces generalizability (external validity) and may result in risk of estimation bias.

Further randomized prospective knee-specific clinical trials would help to additionally confirm these HWDS PROMs findings.

## 5. Conclusions

Analysis of the H-Wave^®^ PROMs data collected sequentially over 4 years suggested positive benefits for cKP patients. At least 20% pain relief or greater was reported in 86% of cKP participants, with an almost 3-point (2.96) decrease following treatment (0–10 NRS scale). Participants reported function/ADL improvement in 96%, while performance of working patients improved in 85%. Additionally, use of pain medication was stopped or decreased in 70%, with sleep improving in 55% of knee injury patients (Figure S2). Almost all cKP patients indicated met or exceeded expectations (96.53%), satisfaction with service (99.55%), and confidence in device use (99.64%). Outcomes for both subgroups, knee injury and degeneration, were very similar to those of all knee conditions. Longer device use seemed to be predictive of greater pain relief, while longer pain chronicity slightly reduced HWDS’ effectiveness. Despite many methodological limitations, which limit causal inference and preclude broader clinical recommendations, this unique form of ES appears to be promising for refractory cKP, suggesting the need for further higher-quality randomized controlled trials for more specific indications.

## Figures and Tables

**Table 1:** Characteristics of H-Wave^®^ device stimulation (HWDS) intervention cohort.

Characteristics	Proportion/Mean ± Standard Deviation		
	All Knee Disorders	Knee Injury	Knee Degeneration
Gender			
Male	599 (52.41%)	526 (53.40%)	73 (58.87%)
Female	544 (47.59%)	459 (46.60%)	51 (41.13%)
Age when injured	47.96 ± 11.59 years	47.66 ± 11.55 years	52.42 ± 10.72 years
Age when treated with HWDS	48.82 ± 11.61 years	48.50 ± 11.56 years	53.47 ± 10.80 years
Duration of pain chronicity	310.85 ± 179.25 days	306.78 ± 178.09 days	380.42 ± 199.22 days
Duration of HWDS usage	93.33 ± 65.55 days	93.15 ± 65.80 days	92.80 ± 66.80 days

**Table 2:** Analysis of primary and secondary outcome measures. * Demonstrates statistical significance (*p* < 0.05).

Outcome Measures		Parameter	All Knee Disorders		Knee Injury		Knee Degeneration	
			Value	*p*-Value	Value	*p*-Value	Value	*p*-Value
**Primary Outcome Measures**	**Pain**	Pre-treatment score	1130 patients (7.06 ± 1.98)	<0.0001 * (difference)	974 patients (7.09 ± 1.95)	<0.0001 * (difference)	123 patients (6.85 ± 2.11)	<0.0001 * (difference)
		Post-treatment score	1127 patients (4.07 ± 2.14)		972 patients (4.07 ± 2.12)		122 patients (4.14 ± 2.25)	
		Difference	1125 patients (2.96 ± 1.77)		971 patients (2.99 ± 1.76)		122 patients (2.72 ± 1.74)	
	**Function/ADL**	Yes	1052 patients (96.51%)	<0.0001 *	907 patients (96.70%)	<0.0001 *	118 patients (98.33%)	<0.0001 *
		No	38 patients (3.49%)		31 patients (3.30%)		2 patients (1.67%)	
	**Medication Usage**	Eliminated	106 patients (13.15%)	<0.0001 * (decreased + eliminated vs. no effect)	95 patients (13.69%)	<0.0001 * (decreased + eliminated vs. no effect)	12 patients (14.81%)	=0.0008 * (decreased + eliminated vs. no effect)
		Decreased	457 patients (56.70%)		393 patients (56.63%)		43 patients (53.09%)	
		No effect	243 patients (30.15%)		206 patients (29.68%)		26 patients (32.10%)	
	**Sleep Improvement**	Yes	630 patients (55.12%)	Not significant	545 patients (55.33%)	*p* = 0.0005	68 patients (54.84%)	Not significant
		No	513 patients (44.88%)		440 patients (44.67%)		56 patients (45.16%)	
**Secondary Outcome Measures**	**Work performance**	Yes	358 patients (84.63%)	<0.0001 *	315 patients (87.74%)	<0.0001 *	36 patients (85.71%)	<0.0001 *
		No	65 patients (15.37%)		44 patients (12.26%)		6 patients (14.29%)	
	**Preference for HWDS** **compared to prior treatment**	More	743 patients (68.04%)	<0.0001 * (HWDS vs. prior treatments)	654 patients (69.28%)	<0.0001 * (HWDS vs. prior treatments)	78 patients (66.10%)	<0.0001 * (HWDS vs. prior treatments)
		Same	328 patients (30.04%)		277 patients (29.34%)		36 patients (30.51%)	
		Less	21 patients (1.92%)		13 patients (1.38%)		4 patients (3.39%)	
	**Patient expectation**	Exceeded	382 patients (34.92%)	<0.0001 * (exceeded or met vs. fallen short)	334 patients (35.46%)	<0.0001 * (exceeded or met vs. fallen short)	39 patients (32.50%)	<0.0001 * (exceeded or met vs. fallen short)
		Met	674 patients (61.61%)		578 patients (61.36%)		77 patients (64.17%)	
		Fallen Short	38 patients (3.47%)		30 patients (3.18%)		4 patients (3.33%)	
	**Patient satisfaction on service**	Excellent	951 patients (85.37%)	<0.0001 * (excellent or satisfactory vs. poor)	825 patients (86.12%)	<0.0001 * (excellent or satisfactory vs. poor)	100 patients (81.97%)	<0.0001 * (excellent or satisfactory vs. poor)
		Satisfactory	158 patients (14.18%)		130 patients (13.57%)		21 patients (17.21%)	
		Poor	5 patients (0.45%)		3 patients (0.31%)		1 patient (0.82%)	
	**Patient confidence on device use**	Excellent	890 patients (80.04%)	<0.0001 * (excellent or satisfactory vs. poor)	775 patients (81.07%)	<0.0001 * (excellent or satisfactory vs. poor)	98 patients (80.33%)	<0.0001 * (excellent or satisfactory vs. poor)
		Satisfactory	218 patients (19.60%)		178 patients (18.62%)		23 patients (18.85%)	
		Poor	4 patients (0.36%)		3 patients (0.31%)		1 patient (0.82%)	

**Table 3:** Effect of treatment duration on outcome measures.

Outcome Measures	Parameter	Trial Period	Early Treatment Period	Late Treatment Period
**Pain relief (pre-/post-treatment score difference)**	Average ± SD	2.61 ± 1.63	2.78 ± 1.77	3.28 ± 1.80
	95% interval	(2.43, 2.79)	(2.57, 2.98)	(3.13, 3.44)
	Sample size	315	293	518
**Medication usage (%)**	Eliminated	7.91	13.94	15.67
	Decreased	60.00	50.48	58.22
	No effect	32.09	35.58	26.11
**Sleep improvement (%)**	Yes	49.37	53.18	59.73
	No	50.63	46.82	40.27
**Work performance (%)**	Yes	83.96	80.00	87.62
	No	16.04	20.00	12.38

## Data Availability

The datasets generated during and/or analyzed during the current study are not publicly available but are available from the corresponding author upon reasonable request.

## Supplementary Materials

The following supporting information can be downloaded at https://www.mdpi.com/article/10.3390/medicina62010075/s1, Figure S1: H-Wave^®^ outcome and usage questionnaire; Figure S2: Summary of H-Wave^®^ device stimulation reported outcomes for (A) all knee disorders, (B) knee injury, and (C) knee degeneration; Table S1: Summary of missing data across all groups.

## Abbreviations

The following abbreviations are used in this manuscript:

cKP	Chronic knee pain
PROMS	Patient-reported outcome measures
HWDS	H-Wave^®^ device stimulation
ADL	Activities of daily living
cLBP	Chronic low back pain
cSP	Chronic shoulder pain
ES	Electrical stimulation
PEMF	Pulsed electromagnetic field therapy
IFC	Interferential current
IFT	Interferential therapy
TENS	Transcutaneous electrical nerve stimulation
NMES	Neuromuscular electrical stimulation
PROs	Patient-reported outcomes
NQF	National Quality Forum
PHI	Protected health information
ACL	Anterior cruciate ligament
MCL	Medial cruciate ligament
LCL	Lateral cruciate ligament
PCL	Posterior cruciate ligament
NRS	Numeric Rating Scale
MCID	Minimal clinically important difference
OA	Osteoarthritis
MCAR	Missing completely at random
MAR	Missing at random
