# Supplementary material for: H-Wave® Device Stimulation for Chronic Knee Pain Disorders: A Patient-Reported Outcome Measures Observational Study
Source: Medicina (Kaunas). 2025 Dec 30;62(1):75. doi: 10.3390/medicina62010075 (PMC12843412; doi:10.3390/medicina62010075)
Supplement: Supplementary file 1 [file medicina-62-00075-s001.zip › medicina-4038042-supplementary.pdf]

Supplementary Materials:

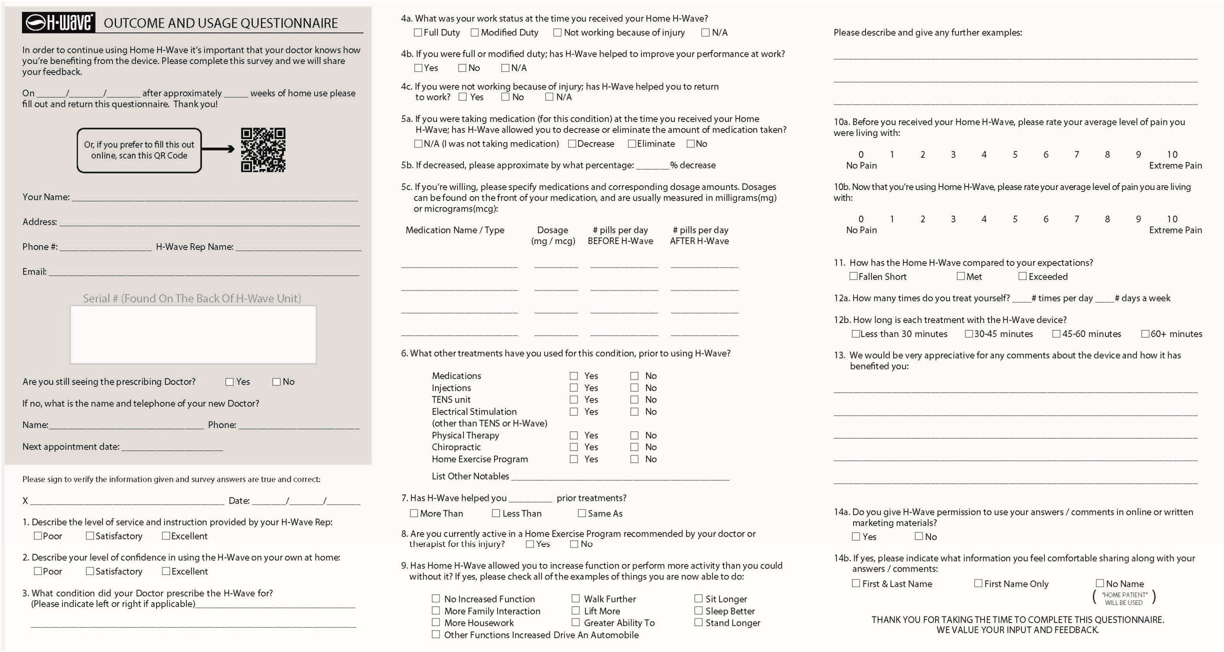

H-Wave OUTCOME AND USAGE QUESTIONNAIRE

In order to continue using Home H-Wave it's important that your doctor knows how you're benefiting from the device. Please complete this survey and we will share your feedback.

On ______/______/______ after approximately _____ weeks of home use please fill out and return this questionnaire. Thank you!

Or, if you prefer to fill this out online, scan this QR Code

Your Name: ______________________

Address: ______________________

Phone #: ______________ H-Wave Rep Name: ______________

Email: ______________________

Serial # (Found On The Back Of H-Wave Unit)

Are you still seeing the prescribing Doctor? ☐ Yes ☐ No

If no, what is the name and telephone of your new Doctor?

Name: ______________ Phone: ______________

Next appointment date: ______________

Please sign to verify the information given and survey answers are true and correct:

X ______________________ Date: ______/______/______

1. Describe the level of service and instruction provided by your H-Wave Rep:
☐ Poor ☐ Satisfactory ☐ Excellent

2. Describe your level of confidence in using the H-Wave on your own at home:
☐ Poor ☐ Satisfactory ☐ Excellent

3. What condition did your Doctor prescribe the H-Wave for?
(Please indicate left or right if applicable) ______________________

4a. What was your work status at the time you received your Home H-Wave?
☐ Full Duty ☐ Modified Duty ☐ Not working because of injury ☐ N/A

4b. If you were full or modified duty; has H-Wave helped to improve your performance at work?
☐ Yes ☐ No ☐ N/A

4c. If you were not working because of injury; has H-Wave helped you to return to work? ☐ Yes ☐ No ☐ N/A

5a. If you were taking medication (for this condition) at the time you received your Home H-Wave; has H-Wave allowed you to decrease or eliminate the amount of medication taken?
☐ N/A (I was not taking medication) ☐ Decrease ☐ Eliminate ☐ No

5b. If decreased, please approximate by what percentage: ________% decrease

5c. If you're willing, please specify medications and corresponding dosage amounts. Dosages can be found on the front of your medication, and are usually measured in milligrams(mg) or micrograms(mcg):

Medication Name / Type	Dosage (mg / mcg)	# pills per day BEFORE H-Wave	# pills per day AFTER H-Wave
____________	______	______	______
____________	______	______	______
____________	______	______	______
____________	______	______	______

6. What other treatments have you used for this condition, prior to using H-Wave?

Medications	☐ Yes	☐ No
Injections	☐ Yes	☐ No
TENS unit	☐ Yes	☐ No
Electrical Stimulation (other than TENS or H-Wave)	☐ Yes	☐ No
Physical Therapy	☐ Yes	☐ No
Chiropractic	☐ Yes	☐ No
Home Exercise Program	☐ Yes	☐ No

List Other Notables ______________________

7. Has H-Wave helped you __________ prior treatments?
☐ More Than ☐ Less Than ☐ Same As

8. Are you currently active in a Home Exercise Program recommended by your doctor or therapist for this injury? ☐ Yes ☐ No

9. Has Home H-Wave allowed you to increase function or perform more activity than you could without it? If yes, please check all of the examples of things you are now able to do:

☐ No Increased Function ☐ Walk Further ☐ Sit Longer
☐ More Family Interaction ☐ Lift More ☐ Sleep Better
☐ More Housework ☐ Greater Ability To ☐ Stand Longer
☐ Other Functions Increased Drive An Automobile

Please describe and give any further examples:

____

10a. Before you received your Home H-Wave, please rate your average level of pain you were living with:

0 1 2 3 4 5 6 7 8 9 10
No Pain — Extreme Pain

10b. Now that you're using Home H-Wave, please rate your average level of pain you are living with:

0 1 2 3 4 5 6 7 8 9 10
No Pain — Extreme Pain

11. How has the Home H-Wave compared to your expectations?
☐ Fallen Short ☐ Met ☐ Exceeded

12a. How many times do you treat yourself? ____# times per day ____# days a week

12b. How long is each treatment with the H-Wave device?
☐ Less than 30 minutes ☐ 30-45 minutes ☐ 45-60 minutes ☐ 60+ minutes

13. We would be very appreciative for any comments about the device and how it has benefited you:

____

14a. Do you give H-Wave permission to use your answers / comments in online or written marketing materials?
☐ Yes ☐ No

14b. If yes, please indicate what information you feel comfortable sharing along with your answers / comments:
☐ First & Last Name ☐ First Name Only ☐ No Name ("HOME PATIENT" WILL BE USED)

THANK YOU FOR TAKING THE TIME TO COMPLETE THIS QUESTIONNAIRE.
WE VALUE YOUR INPUT AND FEEDBACK.

Figure S1. H-Wave® outcome and usage questionnaire.

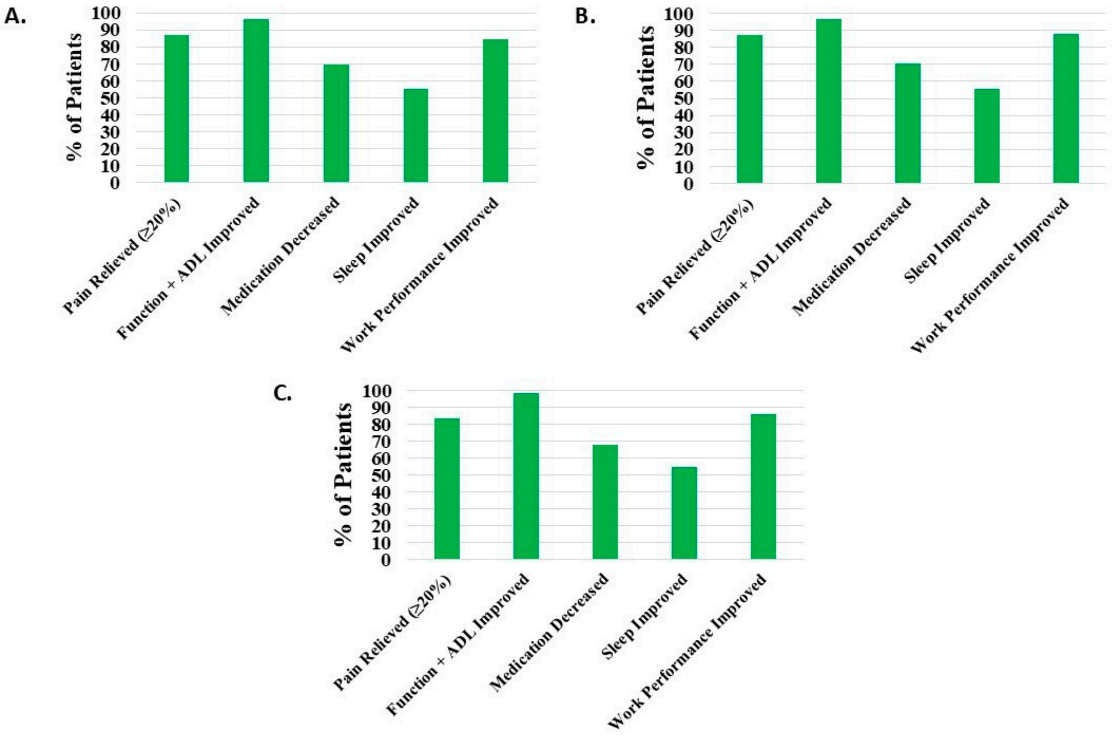

Figure S2. Summary of H-Wave® device stimulation reported outcomes for (**A.**) All Knee disorders, (**B.**) Knee injury and (**C.**) Knee degeneration.

Table S1. Summary of missing data across all groups

Outcome Measure	Missing Data		
	**All Knee Disorders**	**Knee Injury**	**Knee Degeneration**
Function/ADL Improvement	53	40	4
Medication Usage	337	291	43
Work Status and Performance	60	50	5
Preference for HWDS	51	41	6
Patient Expectations	49	43	4
Patient Satisfaction with Service	29	27	2
Patient Confidence in Device Use	31	29	4
